# Determining Ion‐Pair Binding Affinities of Heteroditopic Receptor Systems

**DOI:** 10.1002/chem.202402844

**Published:** 2024-10-16

**Authors:** Andrew Docker, Hui Min Tay

**Affiliations:** ^1^ Yusuf Hamied Department of Chemistry University of Cambridge Lensfield Road Cambridge UK; ^2^ Department of Chemistry Chemistry Research Laboratory University of Oxford Mansfield Road Oxford OX1 3TA UK

**Keywords:** Ion-Pair Recognition, Heteroditopic Receptors, Chalcogen Bonding

## Abstract

Determining ion‐pair affinities in heteroditopic receptor systems presents a persistent and significant challenge. The plethora of technical and experimental problems implicated in measuring ion‐pair affinities have encouraged the use of several expedient experimental practices as a means of characterising ion‐pair recognition behaviour. Exploiting a model heteroditopic receptor system, we interrogate the reliability of these methods and demonstrate that these commonly used techniques can be highly questionable and without extreme care can lead to incorrect conclusions.

## Graphical abstract

Determining ion‐pair affinities in heteroditopic receptor systems presents a persistent and significant challenge. Due to experimental and analytical complexity several expedient techniques to characterise ion‐pair binding behaviour have been widely adopted. Here we experimentally interrogate these methods and reveal serious problems with their uncritical application and interpretation.

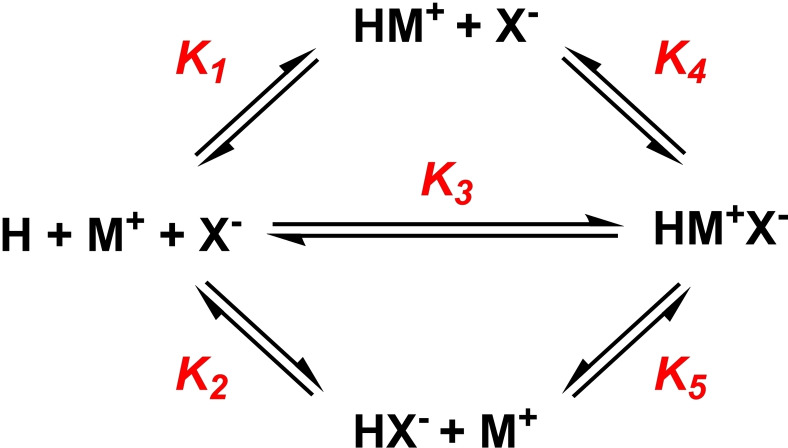

## Introduction

Charged species feature indispensably in innumerable aspects of biological function, environmental processes and chemical industry. It is therefore of little surprise research into the development of host systems capable of strong and selective molecular recognition of ions has continued to stimulate interest.[^1^, ^2^, ^3^] As such, a vast library of receptors now exist targeting a wide array of cation or anion guests exploring various strategies such as selection of non‐covalent interaction type mediating binding behaviour,[^4^, ^5^, ^6^, ^7^, ^8^, ^9^] or molecular topology as a means of generating preorganised recognition environments.[^10^, ^11^, ^12^, ^13^, ^14^, ^15^, ^16^, ^17^, ^18^, ^19^, ^20^] Heteroditopicity,[^21^, ^22^, ^23^] or the integration of recognition sites for both cations and anions, ion‐pairs, in the same receptor architecture has demonstrated significant potential to raise the bar in terms of host‐guest affinity and selectivity profiles relative to their monotopic analogues.[^24^, ^25^, ^26^, ^27^, ^28^, ^29^, ^30^, ^31^, ^32^, ^33^] This modulation of binding is typically attributed to cooperativity, or the mutual influence of co‐bound ions on ion‐pair recognition behaviour. Regardless of receptor type, the quantitative analysis of supramolecular association remains a fundamental metric to compare and inform ion‐host design. Typically, this is achieved through supramolecular titration methods, wherein the perturbation of a physical parameter is measured as a function of host/guest concentrations and appropriately compared and fitted to binding models to determine association constants (*K_a_*), amongst other information.^[34]^ Common methods of monitoring the physical parameter include spectroscopy, such as UV‐Vis and NMR or thermodynamic measurements such as isothermal titration calorimetry (ITC). Of these, ^1^H NMR titration experiments are particularly popular, presumably due to convenience and structural information of the host‐guest complex afforded by the technique. However, without careful consideration of the experimental conditions and chemical properties of the species involved, effects unrelated to that of the binding event including concentration and ionic strength may compromise data collection and obfuscate interpretation.^[35]^ In obtaining quantitative measures for ion‐pair affinities, the existence of multiple equilibria *e. g*. inherent ion‐pairing of the guest ions or the host binding to a single ion presents a considerable challenge. To date, attempts to determine ion‐pair affinities typically rely on application of simpler binding models, which when uncritically examined yield satisfactory results. Indeed, it is often argued that whilst these methods may not be quantitatively reliable, they allow for qualitative descriptors of binding affinities and selectivities in heteroditopic ion‐pair receptors. Furthermore, the often neglected role of solvent can present further problems in these studies. In the field of anion recognition, it has been demonstrated that even fractional aqueous content in the solvent medium almost invariably confers “Hofmeister order”, wherein anion binding selectivity profiles are dictated by hydration enthalpies, *i. e*. the less solvated the anion the more strongly it is bound.^[36]^ Whilst now being recognised as an important factor in anion binding, its consideration in ion‐pair recognition has to the best of our knowledge been absent. Exploiting a chalcogen bonding (ChB) heteroditopic receptor we sought to investigate these common methods of determining ion‐pair affinities, in which we highlight challenges associated with the application and interpretation of results from these methods.

## Results and Discussion

### Discussion Concerning the Measurement of Ion‐Pair Association Constants

**Figure 1:**
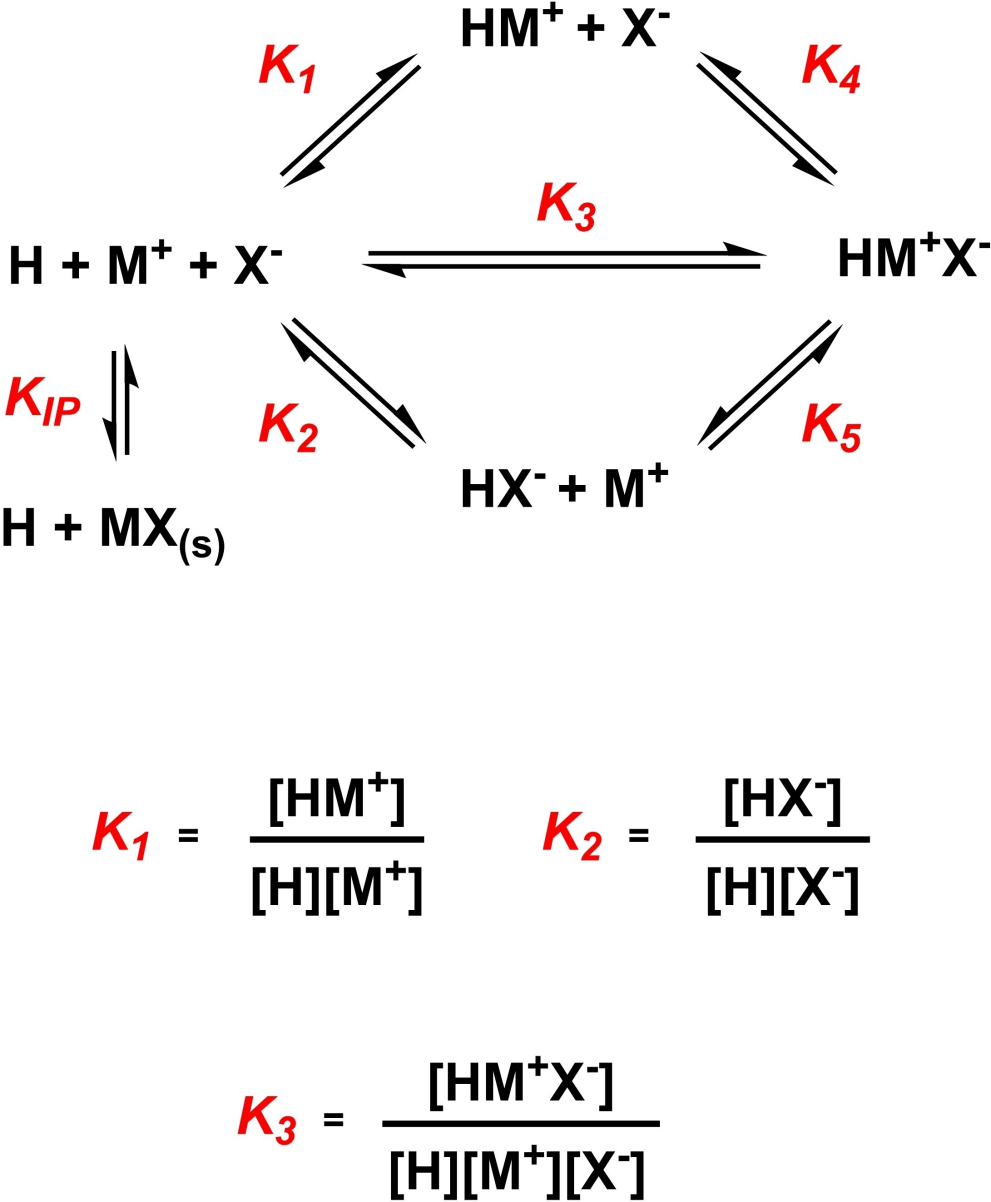
Schematic showing possible equilibria associated with ion‐pair binding.

When considering the ion binding properties of a host receptor (H) to either a cation (M^+^) or anion (X^−^), the equilibria and therefore the association constant, *K_1_* or *K_2_* respectively, may be easily formulated (Figure 1). Applying a similar treatment when considering the ‘ion‐pair’ affinity *i. e*. the simultaneous binding of M^+^ and X^−^ by H generates a comparable expression (*K_3_*) and in general this is understood as the measure of ‘ion‐pair affinity’. Regardless, the ability to determine any association constant relies on the accurate calculation of the concentration of the host/guest complex in question. However, for ion‐pair recognition this is complicated by the potential existence of multiple other species in equilibria which may be practically indistinguishable from the complex of interest and consequently challenging to disentangle. Therefore, as an expediency generally two methods of ion‐pair affinity measurements appear the most popular; i) comparison of an ion affinity in the presence and absence of a counterion (*i. e*. comparing *K_1_* and *K_5_* or *K_2_* and *K_4_*) here called *enhancement* studies or ii) direct addition of the ion‐pair M^+^X^−^ to a solution of H in an attempt to measure *K_3_* here called *direct* studies. Seminal work by Roelens and Francesconi[^37^, ^38^] have elegantly demonstrated these procedures can suffer major inconsistencies from the neglection of competing equilibria and other technical aspects *e. g*. in treatment i) the association constants for the ternary HM^+^X^−^ complex and binary HM^+^ or HX^−^ complexes are incommensurate, due to their differing dimensionality. Notwithstanding these technical issues, the situation in which treatment i) can be potentially justified is the limiting case in which the affinity of H for M^+^ is sufficiently large (*ca. K_a_* >10^4^) such that HM^+^ can be regarded as a singular chemical entity and the concentration of this species is not perturbed by a X^−^ titrant, or *vice versa*. Nonetheless, it should be stressed that those values obtained through this method would serve as a means of comparison within the data set as they are not sufficient to determine genuine free energy changes associated with the binding event.[^39^, ^40^] In the case of treatment ii) the experimental data obtained by the direct method is usually fitted to a 1 : 1 association model, evidently contradictory to the three reagent species of the HM^+^X^−^ complex. Indeed, conceivably these systems would be most appropriately modelled by a heterotropic 1 : 1 : 1 binding stoichiometry model. Furthermore, heteroditopic receptors almost invariably function as competent binders for both cations or anions individually, such that during the course of these titration experiments it may be unclear that the changing physical parameter being measured is a result of the HM^+^X^−^ complex or the HM^+^/HX^−^ complexes. The direct ion‐pair addition in treatment ii) also presents problems from an experimental perspective. To perform direct ion‐pair titration experiments organic aqueous solvent mixtures are typically employed to solubilise the M^+^X^−^ titrant. Whilst the role of aqueous solvent media has been well documented in the field of anion recognition, it appears that solvent effects including ‘Hofmeister order’ in ion‐pair recognition studies are seldom considered. To experimentally explore the reliability of these traditionally excepted methods we selected the chalcogen bonding (ChB) heteroditopic receptor, **1⋅ChB^PFP^**, as a convenient ion‐pair receptor model system with an extensively investigated and characterised binding behaviour. Due to the complex nature of the equilibria and as a means of tracking the potentially numerous ionic species involved, ^1^H NMR studies were selected as the principal means of structurally interrogating and quantifying ion‐pair binding behaviour.

### *Enhancement* Ion‐Pair Binding Studies

**Figure 2:**
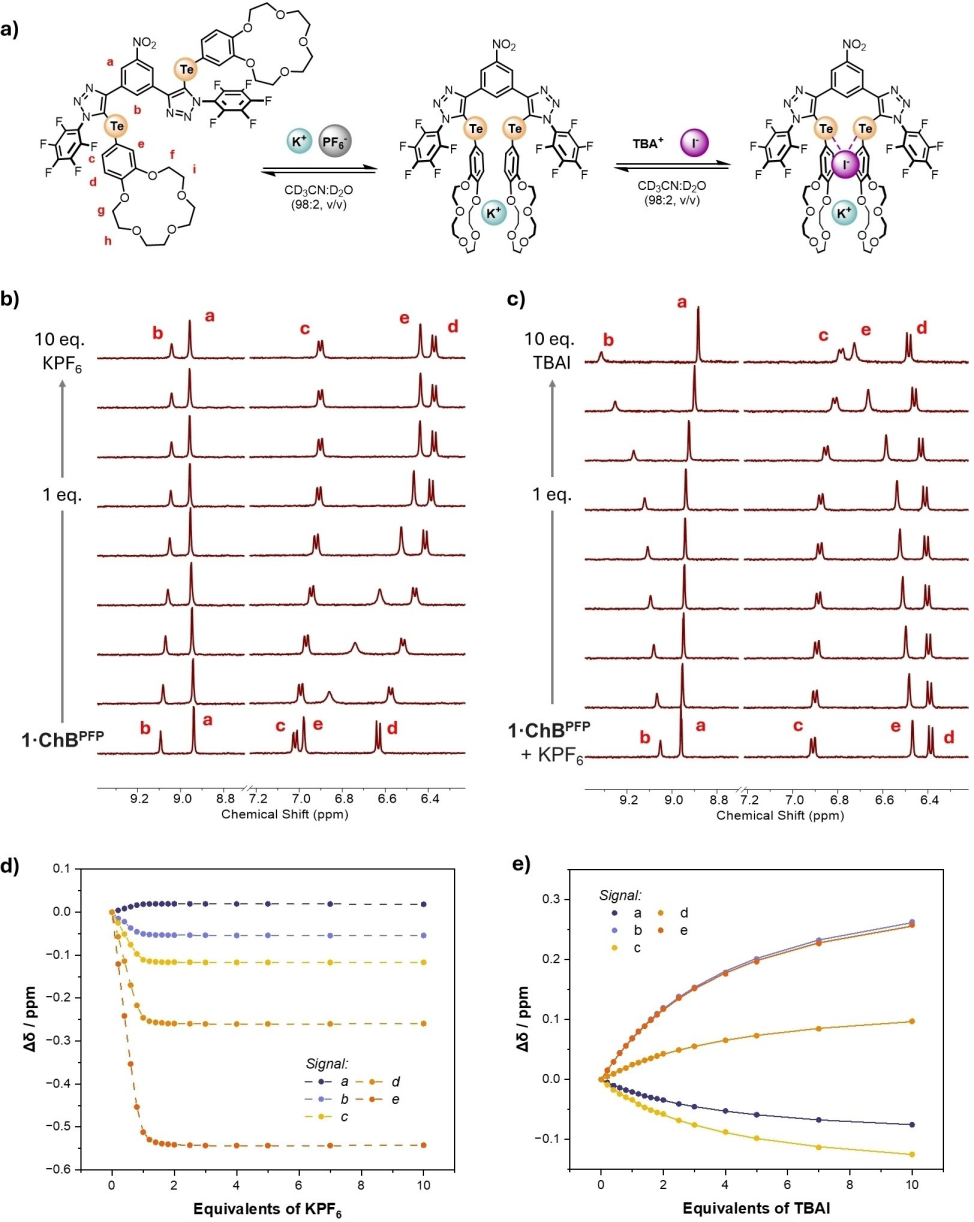
a) Ion‐Pair **1⋅ChB^PFP^** binding equilibria. Truncated spectra from ^1^H NMR titration experiments of **1⋅ChB^PFP^** with b) KPF_6_ and c) TBAI in the presence of one equivalent of KPF_6_ (98 : 2 CD_3_CN/D_2_O (v/v), 500 MHz, 298 K). Binding isotherms; d) for KPF_6_ titration e) for TBAI titration in the presence of 1 equivalent of KPF_6_ where circles represent experimental data, dotted lines are a visual aid and solid lines show fitted isotherms.

The receptor selected, **1⋅ChB^PFP^**, consists of a 3,5‐bis‐tellurotriazole nitro‐benzene central scaffold functionalised with electron‐deficient perfluorophenyl substituents and benzo‐15‐crown‐5 (B15 C5) appended telluro‐triazoles.[^41^, ^42^] Wherein, the pendant B15 C5 units are capable of complexing alkali metal (K^+^, Rb^+^, Cs^+^) or ammonium (NH_4_^+^) cations *via* an intramolecular sandwich complex in a 1 : 1 **1⋅ChB^PFP^**:M^+^ stoichiometry. This preorganisation and electronic activation[^43^, ^44^] of the Te‐based ChB donors serves to switch on the anion binding potency and facilitates the formation of a ternary ion‐pair complex of 1 : 1 : 1 **1⋅ChB^PFP^**:M^+^:X^−^ stoichiometry. Of the aforementioned cations, potassium was selected as the model for the study due to its strong affinity for B15 C5 and wide ranging solubility. To minimise the effects of exogenous ion‐pairing and allow for the direct ion‐pair titration method, acetonitrile‐aqueous solvent mixtures were selected as the medium for the titration investigations. Initially, the potassium cation recognition capabilities of **1⋅ChB^PFP^** were investigated *via* ^1^H NMR titration studies conducted by adding increasing equivalents of K^+^ as the non‐coordinating PF_6_^−^ salt to a 98 : 2 CD_3_CN/D_2_O (v/v) solution of the receptor (Figure 2a). The addition of the potassium cation induced significant perturbations and broadening of the B15 C5 methylene and aromatic signals consistent with the formation of an intramolecular cofacial K^+^ bis‐B15 C5 sandwich complex.[^45^, ^46^, ^47^] Selecting five well resolved and unobscured proton signals namely; a and b of the central nitro‐benzene unit and c,d,e of the B15 C5 aromatic linker, the visualisation of these chemical shift perturbations (Δδ) as a function of KPF_6_ equivalents reveals a typical isotherm associated with formation of a 1 : 1 **1⋅ChB^PFP^**: K^+^ stoichiometric complex with an affinity of >10^5^ M^−1^. As anticipated, the crown ether signals c, d and e exhibit the largest changes, undergoing dramatic upfield shifts whilst the nitro‐benzene aromatic signals a and b are to a lesser extent shifted downfield and upfield respectively. The capability of **1⋅ChB^PFP^** to bind a KI ion‐pair was investigated by ^1^H NMR anion titration experiments conducted in the presence of equimolar KPF_6_. Wherein addition of an iodide solution, as its tetrabutylammonium (TBA) salt, induced downfield perturbations in signals b, d, e and upfield perturbations in a and c. During the course of iodide addition it was noted the diagnostic features of the K^+^B15 C5 sandwich complex persisted upon anion addition, indicating simultaneous K^+^ and I^−^ complexation. Bindfit analysis^[†]^ of the anion‐induced chemical shift perturbations of signals a, b, c, d and e determined concordant 1 : 1 stoichiometric host/guest association constants in the range of 246–300 M^−1^ (Table 1). Notably, no anion induced chemical shift perturbations were observed in the absence of K^+^ indicating a cobound potassium cation is required for switching on the ChB mediated anion binding potency of **1⋅ChB^PFP^** (estimated upper bound of *K_a_* (I) <10 M^−1^ for **1⋅ChB^PFP^** in the absence of KPF_6_).

**Table 1:** Iodide association constants for **1⋅ChB^PFP^** in the presence of 1 equivalent of KPF_6_ from ^1^H NMR titration experiments (98 : 2 CD_3_CN/D_2_O (v/v), 298 K).

Signal ^[a]^	**a**	**b**	**c**	**d**	**e**
*K_a_* (I^−^)/M^−1 [b]^	289 (±3)	269 (±3)	300 (±4)	246 (±3)	276 (±3)

[a] Bindfit analysis monitoring a specific signal. [b] Errors indicated in parentheses.

Crucially, several factors contribute to the credibility of data obtained by these *enhancement* studies; i) the high stability of the K^+^:**1⋅ChB^PFP^** complex ii) the confirmation of integrity of the K^+^B15 C5 sandwich complex during iodide addition iii) the consistency of *K_a_* (I^−^) values obtained by the fitting of multiple signals. For **1⋅ChB^PFP^** the persistence of the potassium complex is evidenced through diagnostic spectroscopic features, however in the absence of this it would challenging to rule out an exogenous ion‐pairing event (*K_IP_*) wherein perturbations could be due to cation decomplexation.

**Figure 3:**
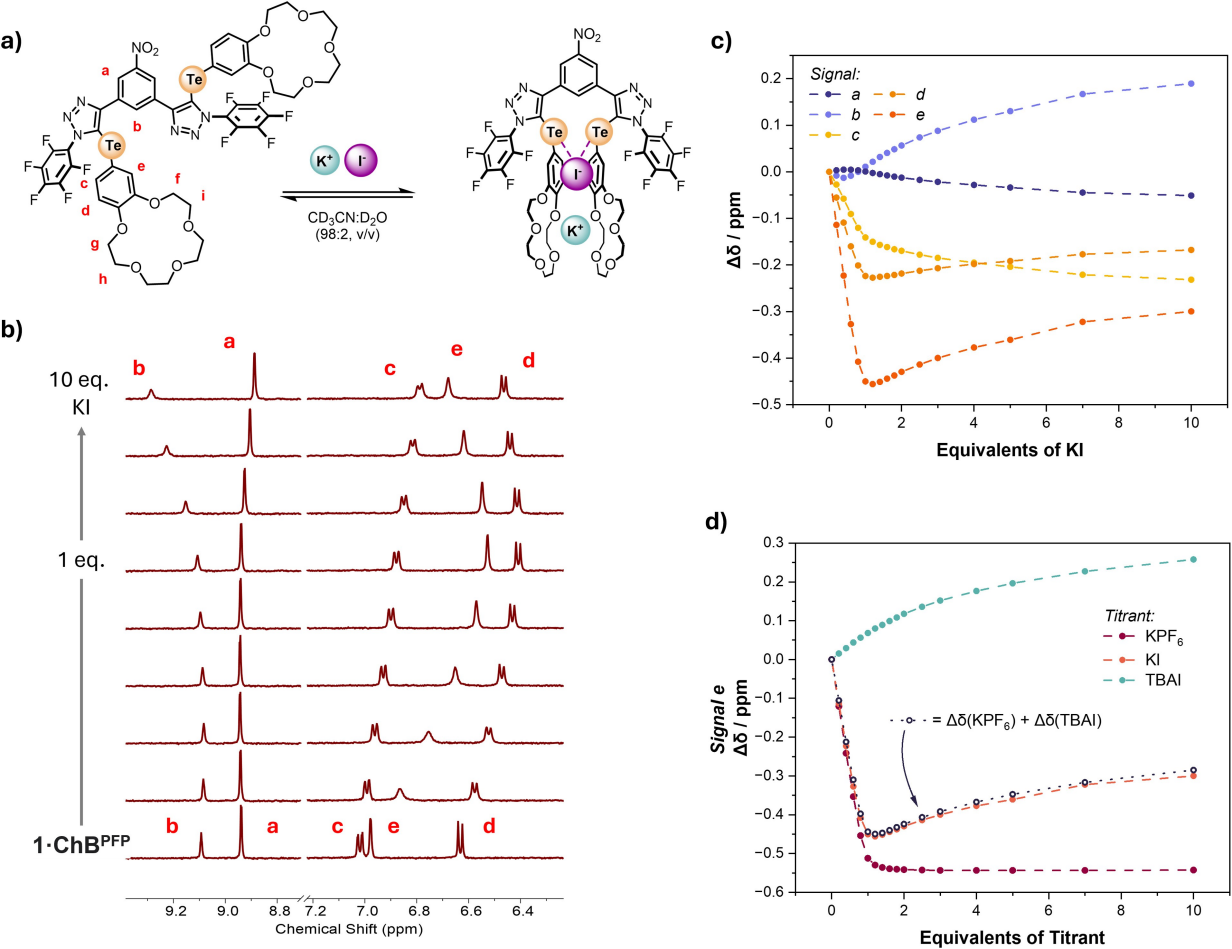
a) Potassium iodide **1⋅ChB^PFP^** binding equilibria. b) Truncated spectra from the direct KI ^1^H NMR titration experiment of **1⋅ChB^PFP^** (98 : 2 CD_3_CN/D_2_O (v/v), 500 MHz, 298 K). c) Binding isotherms for KI titration d) Binding isotherms for signal e with the titrants KPF_6_, KI and TBAI with the combined Δδ profiles of KPF_6_ and TBAI titrations. Circles represent experimental data, dotted lines are a visual aid. Hollow circles represent combined Δδ values.

With the perturbation behaviour of the selected proton signals of **1⋅ChB^PFP^** noted during the course of potassium iodide recognition, attention was directed towards the direct addition of KI (Figure 3a). To this end, a similar ^1^H NMR titration experiment was conducted wherein KI was added directly to a 98 : 2 CD_3_CN/D_2_O (v/v) solution of **1⋅ChB^PFP^**. In contrast to the cation and anion titration experiments, the direct addition of KI reveals a much more complex Δδ profile, signals a, b, c, d and e exhibit inflection points, *i. e*. reversals in chemical shift perturbation direction during the course of the experiment (Figures 3b and c). Furthermore, the number of equivalents of KI at which the inflection point occurs differs for each signal. It is clear from inspecting the direction and magnitude of chemical shift perturbations associated with K^+^ and I^−^ binding that the profile observed during direct KI addition is a combination of those binding events. Indeed, this is especially evident when combining the Δδ profiles for the KPF_6_ and TBAI titrations (Δδ(KPF_6_)+Δδ(TBAI)) which map onto the KI titrant isotherm, a representative example is shown in Figure 3d. It is therefore apparent that during the course of the KI titration the ^1^H NMR spectrum provides evidence for the formation of at least two species; the **1⋅ChB^PFP^**:K^+^ and the **1⋅ChB^PFP^**:K^+^:I^−^ complexes. Whilst perhaps expected, these results serve to illustrate an important point regarding treatment of experimental results obtained from direct ion‐pair titrations. Firstly, notwithstanding the aforementioned technical issues, attempts to fit this data to a 1 : 1 host/guest stoichiometric model are evidently problematic, as the undulating profile of the chemical shift change cannot be sensibly rationalised by a host/guest complex of this stoichiometry. In the case of **1⋅ChB^PFP^**, the opposing direction of chemical shift perturbations associated with cation and anion binding, it is clear that a 1 : 1 stoichiometric model is not appropriate. More generally however, it is important to stress that even if a direct ion‐pair titration affords unidirectional changes in a chemical shift being measured, it is still not correct to apply a 1 : 1 stoichiometric model. For **1⋅ChB^PFP^** it is fortuitous that due to the physical characteristics of the system clear identification of different species present is possible, however, this may not always be the case. For example consider if both K^+^ and I^−^ binding induced chemical shift perturbations in the same direction, identification of the multiple complexes present would become more challenging and superficially consistent with a 1 : 1 host/guest complex.

### *Direct* Ion‐Pair Binding Studies

**Figure 4:**
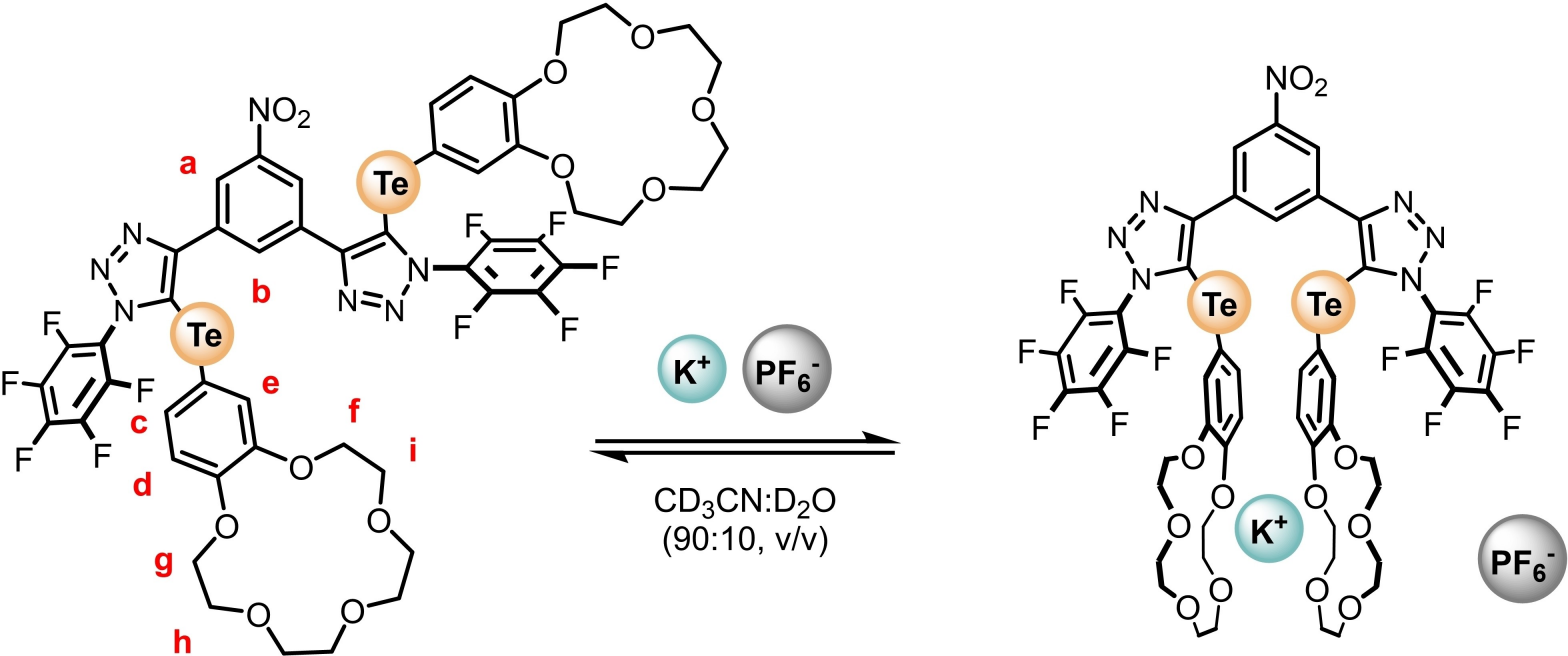
Potassium hexafluorophosphate binding equilibria of **1⋅ChB^PFP^**.

**Figure 5:**
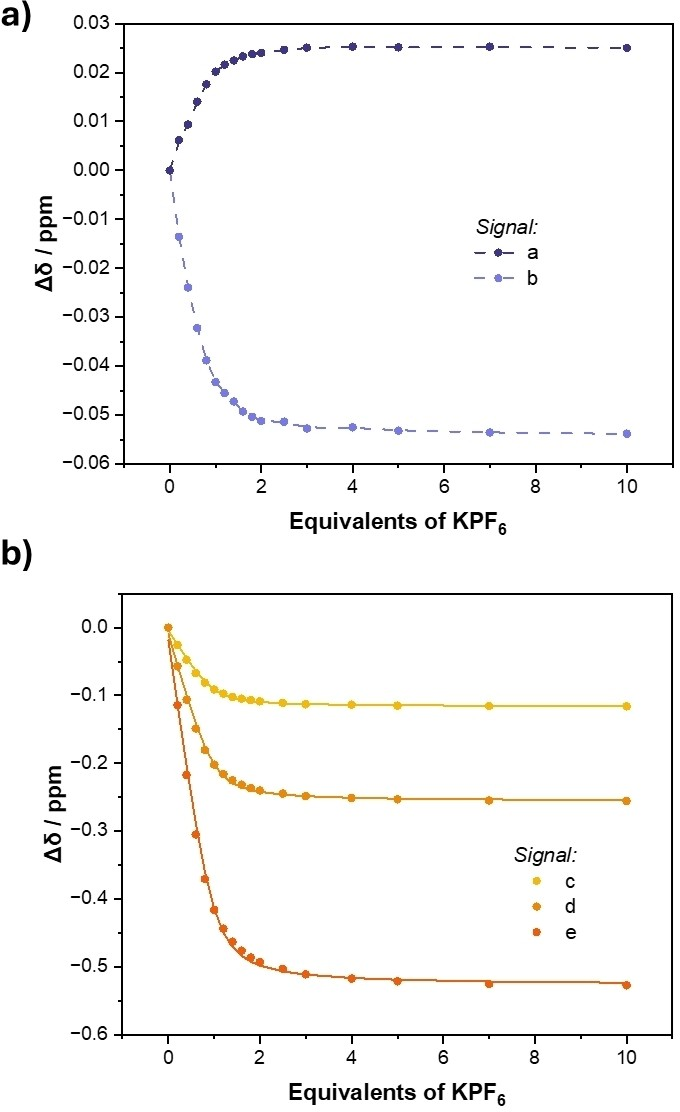
Binding isotherms for KPF_6_ titration with **1⋅ChB^PFP^** monitoring a) signals a and b b) monitoring signals c, d and e. Circles represent experimental data, dotted lines are a visual aid and solid lines show fitted isotherms (90 : 10 CD_3_CN/D_2_O (v/v), 500 MHz, 298 K).

Another popular method of characterising ion‐pair binding properties of heteroditopic receptors include comparing ‘*K_a_* values’ of different salts obtained from *direct* studies, to determine ion‐pair selectivity profiles. To investigate this method we sought to undertake a further series of experiments wherein a range of potassium salts could be titrated, necessitating the use of a solvent medium with a higher aqueous content specifically 90 : 10 CD_3_CN/D_2_O (v/v). To first examine the K^+^ binding properties of **1⋅ChB^PFP^** in this solvent medium, a KPF_6_^1^H NMR titration was conducted (Figure 4). In a similar manner to the analogous experiment conducted in 98 : 2 CD_3_CN/D_2_O (v/v), the diagnostic characteristics of the K^+^ bis‐B15 C5 sandwich complex are observed in addition to chemical shift perturbations of aromatic signals a, b, c, d and e, exhibiting Δδ profiles consistent with a 1 : 1 binding event (Figure 5). Bindfit analysis of c, d and e determined 1 : 1 stoichiometric host/guest association constants of approximately 16,000 M^−1^ (Table 2), an expected diminution in K^+^ affinity relative to the those performed in a lower aqueous content solvent mixture is observed, but importantly still of sufficient magnitude to allow for the determination of potassium complexation induced anion binding enhancement factors.

**Table 2:** Potassium Cation Association Constants for **1⋅ChB^PFP^** from ^1^H NMR Titration Experiments with KPF_6_ (90 : 10 CD_3_CN/D_2_O (v/v), 298 K).

Signal^[a]^	**c**	**d**	**e**
*K_a_* (K^+^)/M^−1 [b]^	15,600 (±2630)	16,600 (±2970)	16,200 (±2840)

[a] Bindfit analysis monitoring a specific signal. [b] Errors indicated in parentheses.

**Figure 6:**
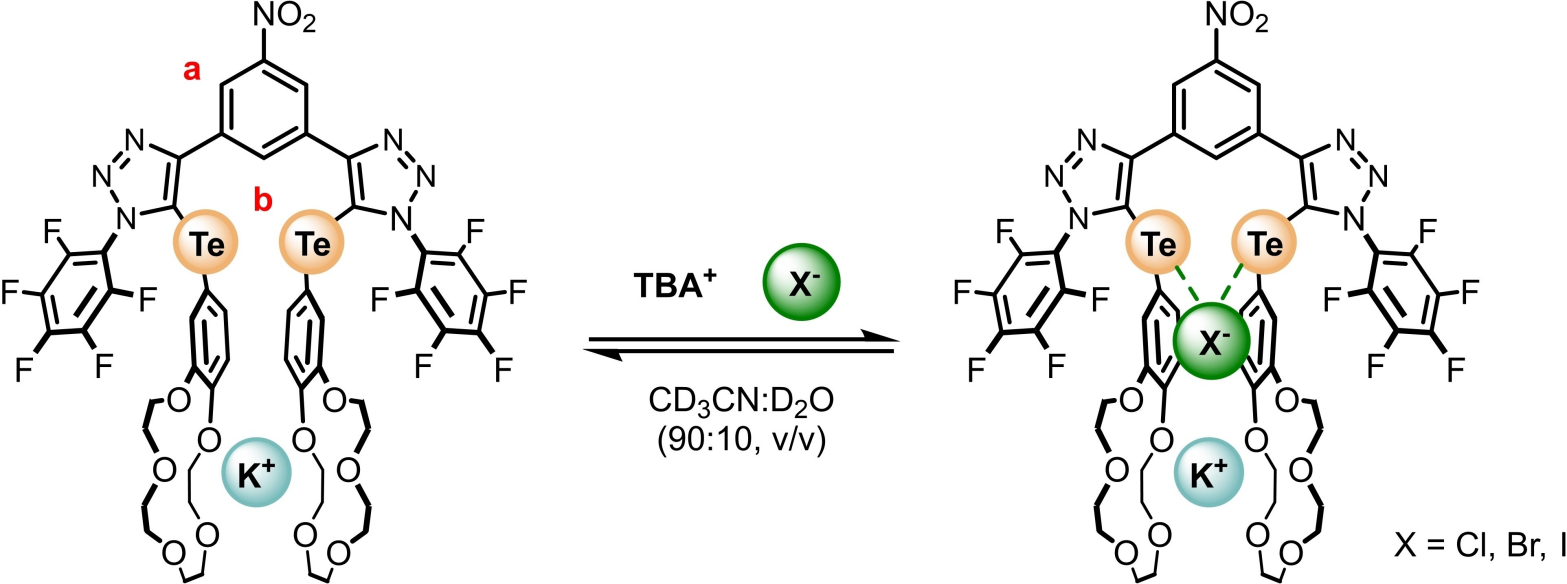
Halide binding equilibria of **1⋅ChB^PFP^** precomplexed with 1 equivalent of KPF_6_.

Accordingly, the potassium halide ion‐pair recognition capabilities were investigated, in which a TBAX (X=I^−^, Br^−^, Cl^−^) solution was added to an equimolar preformed mixture of **1⋅ChB^PFP^** and KPF_6_ (Figure 6). As before, the addition of anion titrant induced the most significant shifts in proton signals proximal to the ChB anion binding cleft. Monitoring signal a determined anion *K_a_* values of 82 M^−1^, 20 M^−1^ and <10 M^−1^ for iodide,^[≠]^ bromide and chloride respectively, summarised in Table 3. As anticipated **1⋅ChB^PFP^** exhibits an attenuated *K_a_* (I^−^) in this solvent mixture value relative to the same experiment performed in 98 : 2 CD_3_CN/D_2_O (v/v) due to the increased solvent competitivity. In wholly organic solvent media anion selectivity profiles are usually governed by anion basicity, for which a trend of *K_a_* (Cl^−^)> *K_a_* (Br^−^)> *K_a_* (I^−^) is expected. However, due to ‘preferential solvation’ of anions by water molecules a ‘Hofmeister order’ is observed, such that the trend is reversed and affinities closely mirror levels of anion solvation.^[48]^

**Table 3:** Anion Association Constants for **1⋅ChB^PFP^** in the presence of 1 equivalent of KPF_6_ from ^1^H NMR Titration Experiments monitoring signal a (90 : 10 CD_3_CN/D_2_O (v/v), 298 K).

Anion ^[a]^	**Cl^−^**	**Br^−^**	**I^−^**
*K_a_* (X^−^)/M^−1 [b]^	<10	20 (±1)	82 (±1)

[a] Anions added as their TBA salts. [b] Errors indicated in parentheses.

**Figure 7:**
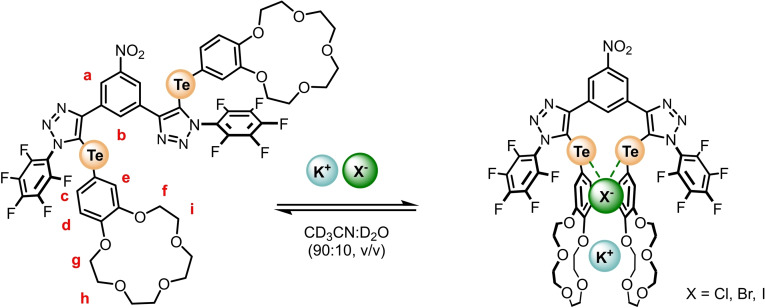
Potassium halide binding equilibria of **1⋅ChB^PFP^**.

**Figure 8:**
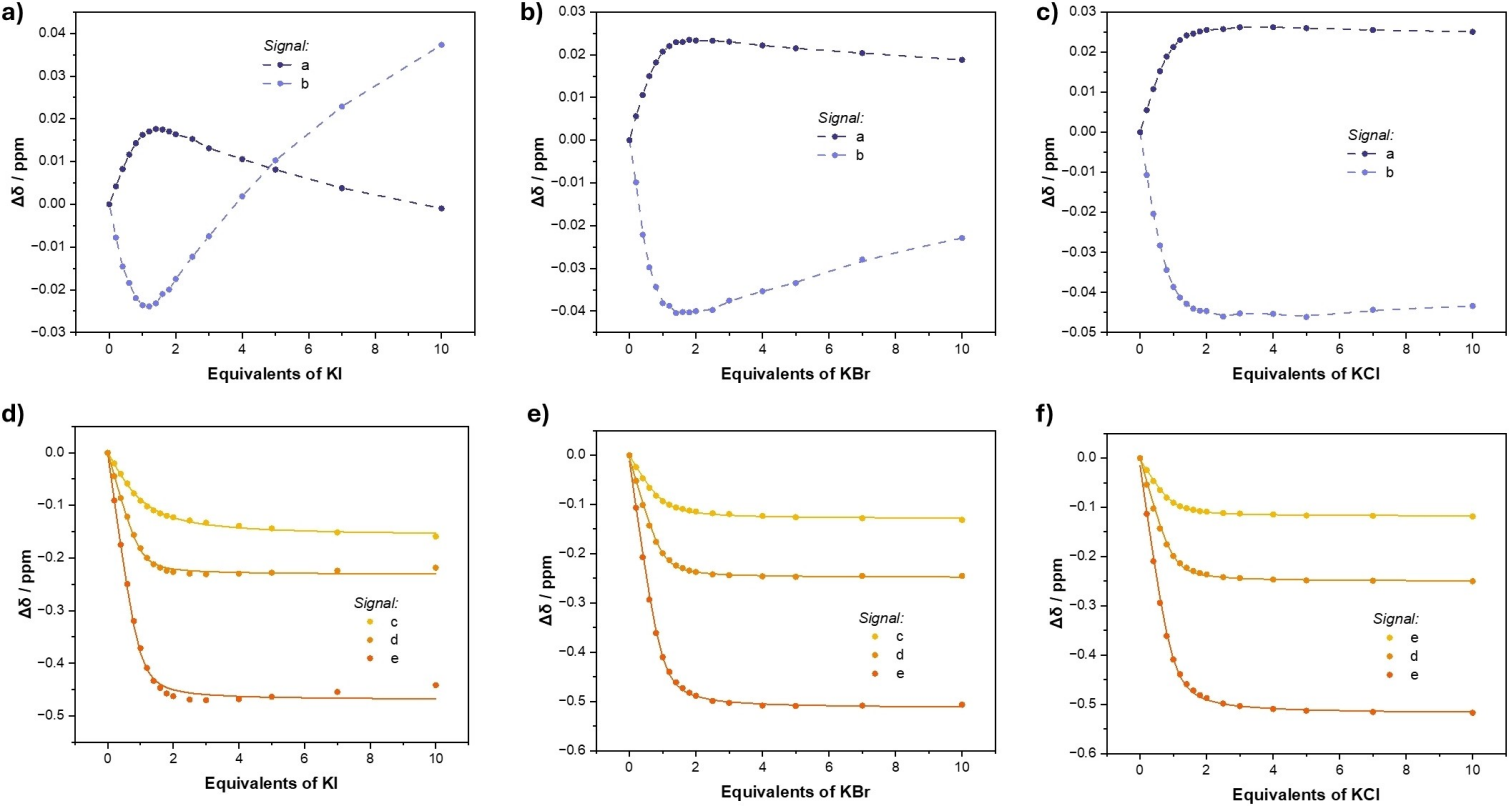
Binding isotherms for potassium halide (KX) titrations with **1⋅ChB^PFP^**. Monitoring signals a and b for a) KI b) KBr c) KCl. Monitoring signals c,d and e for d) KI e) KBr f) KCl. Circles represent experimental data, dotted lines are a visual aid and solid lines show fitted isotherms (90 : 10 CD_3_CN/D_2_O (v/v), 500 MHz, 298 K)

With these results in hand, attention was focused on undertaking the *direct* ion‐pair titration experiments with the corresponding potassium halides; KI, KBr and KCl (Figure 7). For all *direct* potassium halide titrations the ^1^H NMR spectra confirm potassium cation complexation and the observed Δδ profiles for signals a,b,c,d and e are summarised in Figure 8. Inspection of the perturbations of nitro‐benzene signals a and b in the KBr or KI titrations show a complex profile with inflection points in Δδ values at *ca*. 1 equivalent of KI or KBr (Figures 8a–c), for reasons similar to those in the 98 : 2 CD_3_CN/D_2_O (v/v) experiments. In contrast, monitoring of the crown ether aromatic signals c,d and e show Δδ profiles which appear to show a typical 1 : 1 host/guest binding profile (Figures 8d–f). It is important to note, the observation of inflection points in signals a and b and the lack thereof in signals c, d and e is a result of the subtle interplay of at least three factors. Firstly, the magnitude of perturbation for a chemical shift of a specific signal between the free receptor **1⋅ChB^PFP^** and when fully bound to an ion(s). Secondly, the direction of perturbation associated with each binding event *e. g*. K^+^ binding causes a downfield shift in signal a, but I^−^ binding to the **1⋅ChB^PFP^**: K^+^ complex induces an upfield shift in signal a. Thirdly, the relative contributions of chemical shift perturbations by various guest binding events to the overall Δδ observed will be weighted by their individual *K_a_* values. This third factor is easily visualised by comparing the Δδ profiles for signal a and b in the direct KI, KBr and KCl titrations. It is observed that K^+^ binding causes a downfield and upfield shifts in signals a and b respectively, whilst halide anion binding causes the reverse; upfield and downfield shifts in a and b respectively. The decreasing halide affinities of I^−^, Br^−^ and Cl^−^ cause a concomitantly less noticeable influence on these Δδ profiles such that the inflection point for the KBr titration is much less pronounced compared to the KI experiment and is barely visible for the KCl titration. However, due to a combination of the listed factors above, these effects are not observed in the Δδ profiles for the crown ether aromatic signals c, d and e. Indeed, in the absence of other information, unscrutinised examination of the direct ion‐pair ^1^H NMR titrations monitoring signals c,d and e would appear to satisfy a typical 1 : 1 stoichiometric host/guest complex. Furthermore, Bindfit analysis of the data for crown ether aromatic signals c,d and e can determine 1 : 1 stoichiometric host/guest association constants, which upon casual inspection give *K_a_* values generally in the expected range and with acceptable fitting errors, the results of which are summarised in Table 4 and are often considered ion‐pair ‘affinities’ or ‘association constants’.

**Table 4:** Potassium Halide ‘Ion‐Pair Association Constants’ for **1⋅ChB^PFP^** from ^1^H NMR Titration Experiments with KX (90 : 10 CD_3_CN/D_2_O (v/v), 298 K).

KX Titrant	Signal Monitored^[a]^		
	**c**	**d**	**e**
KI	2,940 (±232)	21,400 (±5140)	23,000 (±6000)
KBr	8,590 (±1030)	20,200 (±2828)	19,300 (±2510)
KCl	13,200 (±1590)	19,300 (±3470)	16,500 (±2150)

[a] Bindfit analysis monitoring a specific signal. [b] Errors indicated in parentheses.

**Figure 9:**
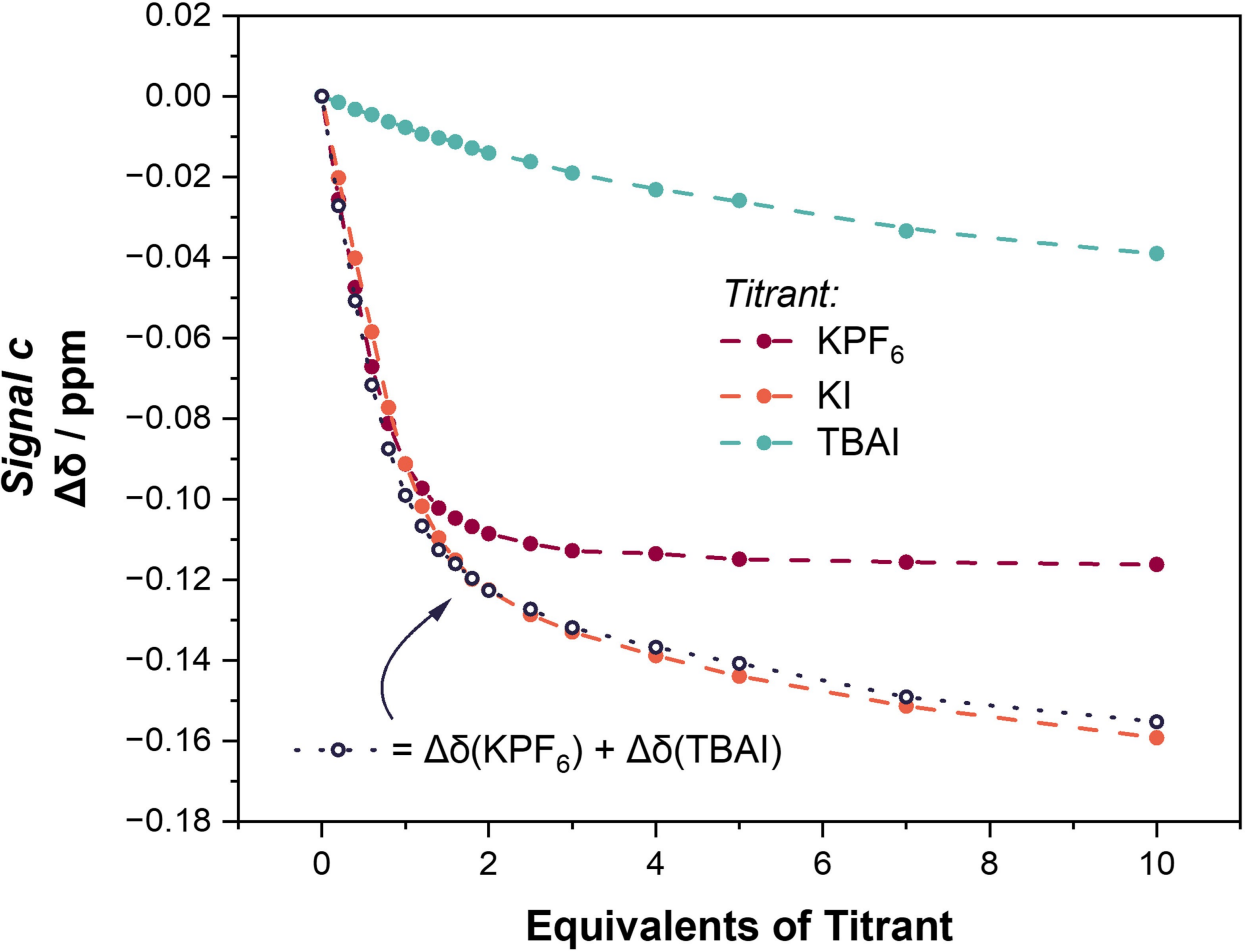
Binding isotherms for signal c with the titrants KPF_6_, KI and TBAI with the combined Δδ profiles of KPF_6_ and TBAI titrations. Circles represent experimental data, dotted lines are a visual aid and the hollow circles represent combined Δδ values.

A survey of the ‘ion‐pair association constants’ in Table 4 show values which span *ca*. 2,900 M^−1^ and 23,000 M^−1^. The modelling of this data by this method, and therefore its interpretation, is clearly problematic. Firstly, the wide range of ‘ion‐pair association constants’ determined by monitoring different signals immediately suggests unreliability, *e. g*. for the direct KI titration a ‘*K_a_* (KI)’ of 2,940 M^−1^ or 23,000 M^−1^ can be obtained by fitting Δδ values for signal c or e respectively. However, by examining all available titration data it is possible to rationalise these apparent values by recognising the perturbation in chemical shifts is composed of multiple species present in equilibrium. For example, consider the anomalously low *K_a_* (KI) value obtained by monitoring signal c during the direct KI titration experiment. It is shown that signal c is perturbed upfield by both K^+^ binding and I^−^ binding to the **1⋅ChB^PFP^**: K^+^ complex. When KI is added directly these affects are roughly additive and the resultant isotherm appears to reach its maximum Δδ value at a larger number of equivalents of KI resulting in a lower determined *K_a_* value (Figure 9). Furthermore, consider the case in which only signal c was available for analysis, on this basis it would be reasonable to argue for **1⋅ChB^PFP^** ‘s impressive ‘KCl selectivity’. This apparent ‘ion‐pair selectivity’ determined by monitoring signal c yields the following; *K_a_* (KCl)=13,100 M^−1^ *K_a_* (KBr)=8,590 M^−1^ and *K_a_* (KI)=2,940 M^−1^ and suggests selectivity for KCl over the other potassium halides. When considering the halide affinities for the **1⋅ChB^PFP^**: K^+^ complex, *K_a_* (Cl^−^)=<10 M^−1^ and *K_a_* (Br^−^)=20 M^−1^ *K_a_* (I^−^)=82 M^−1^ the ‘ion‐pair selectivity’ appears directly contradictory. The inconsistency of this interpretation is further exemplified if the direct KPF_6_ titration is also viewed as a ‘KPF_6_’ ion‐pair affinity measurement which yields a *K_a_* (KPF_6_)=15,600 M^−1^, suggesting **1⋅ChB^PFP^**’s affinity for a potassium salt with a non‐coordinating anion of no measurable affinity for the 1 : 1 **1⋅ChB^PFP^**: K^+^ complex is larger than that of the potassium halide ion‐pairs (I^−^, Br^−^, Cl^−^) where the counteranion is demonstrated to bind.

Furthermore, the role of the solvent also cannot be underestimated, whilst it is demonstrated halide affinities are governed by ‘Hofmeister order’ this also extends to the ion‐pair binding affinities. Comparison of the experimental data for the direct KCl and KPF_6_ titrations and chloride affinity measurements to the **1⋅ChB^PFP^**: K^+^ complex seems to suggest that in this solvent mixture chloride is solvated to such an extent that it is commensurate with hexafluorophosphate in terms of coordinating strength.

## Conclusions

From the results above, it is apparent that data obtained from ion‐pair titration experiments must be treated with extreme care. Through extensive ^1^H NMR cation, anion and ion‐pair titration experiments we have attempted to thoroughly characterise the ion‐pair recognition behaviour of **1⋅ChB^PFP^**. Wherein, we have applied two of the most popular means by which ion‐pair binding of heteroditopic receptors are investigated. In the case of *enhancement* studies we have attempted to outline the requisites for meaningful collection and interpretation of this data, highlighting potential pitfalls when using this method and greater appreciation of its limitation. The investigation into *direct* studies highlights significant problems. An uncritical acceptance of experimental data and application of simplistic binding models can produce misleading and evidently erroneous results. Indeed, it is difficult to conceive of a situation in which a 1 : 1 host/guest stoichiometric binding model for ion‐pair binding can ever be justified and any results obtained are highly questionable. In addition, we also draw attention to how experimental conditions, such as solvent effects and specifically ‘Hofmeister order’ can influence binding studies and induce ‘selectivities’ unrelated to the receptor's properties. It should also be stressed that whilst this study utilises ^1^H NMR titration experiments, these problems are also present in other methods such as UV‐Vis and as such should be considered whenever conducting ion‐pair affinity measurements. Determining ion‐pair affinities present a myriad of practical and analytical problems, it is hoped that the findings of this work will inform future efforts into the field of heteroditopic ion‐pair recognition and encourage a more critical scrutiny of experimental observations.

## Supporting Information Summary

Additional supporting information can be found online in the Supporting Information section at the end of this article.

## Conflict of Interests

The authors declare no conflict of interest.

1

## Supporting information

As a service to our authors and readers, this journal provides supporting information supplied by the authors. Such materials are peer reviewed and may be re‐organized for online delivery, but are not copy‐edited or typeset. Technical support issues arising from supporting information (other than missing files) should be addressed to the authors.

Supporting Information

## Data Availability

The data that support the findings of this study are available in the supplementary material of this article.
