# Supplementary material for: Determining Ion‐Pair Binding Affinities of Heteroditopic Receptor Systems
Source: Chemistry. 2024 Oct 16;30(68):e202402844. doi: 10.1002/chem.202402844 (PMC11618037; doi:10.1002/chem.202402844)
Supplement: Supplementary file 1 — Supporting Information [file CHEM-30-e202402844-s001.pdf]

Chemistry–A European Journal

Supporting Information

Determining Ion-Pair Binding Affinities of Heteroditopic Receptor Systems

Andrew Docker* and Hui Min Tay

Supporting information for

Determining Ion-Pair Binding Affinities of Heteroditopic Receptor Systems

Andrew Docker,*[a] Hui Min Tay,[b]

[a] Yusuf Hamied Department of Chemistry, University of Cambridge, Lensfield Road, Cambridge, CB2 1EW U.K.

[b] Chemistry Research Laboratory, Department of Chemistry, University of Oxford, Mansfield Road, Oxford OX1 3TA, U. K.

Contents

1. Materials and Methods 2
2. ^{1}H NMR Titration Studies 2
   Titrations in 98:2 CD_3CN/D_2O (v/v) 3
   Titrations in 90:10 CD_3CN/D_2O (v/v) 6
3. References 12

1. Materials and Methods

All solvents and reagents were purchased from commercial suppliers and used as received unless otherwise stated. Dry solvents were obtained by purging with nitrogen and then passing through an MBraun MPSP-800 column. H_2O was de-ionized and micro filtered using a Milli-Q ® Millipore machine. Routine NMR spectra were recorded on either a Varian Mercury 300, a Bruker AVIII 400 or a Bruker AVIII 500 spectrometer with ^1H NMR titrations recorded on a Bruker AVIII 500 spectrometer. Tetrabutylammonium (TBA) salts were stored in a vacuum desiccator containing phosphorus pentoxide prior to use. Where mixtures of solvents were used, ratios are reported by volume. Chemical shifts are quoted in parts per million relative to the residual solvent peak. **1·ChB^{PFP}** was synthesised according to literature procedures.[1]

2. ^1H NMR Titration Studies

Titration Protocol: In a typical ^1H NMR anion titration experiment, aliquots of the titrant were added to a solution of **1·ChB^{PFP}**. Spectra were recorded at 0, 0.2, 0.4, 0.6, 0.8, 1.0, 1.2, 1.4, 1.6, 1.8, 2.0, 2.5, 3.0, 4.0, 5.0, 7.0 and 10 equivalents. Solvent mixtures quoted are volume by volume ratios. All titrations were repeated at least twice and below are shown representative examples.

Enhancement Studies:

Host: [**1·ChB^{PFP}**] = 1.0 mM

Pre-complexed KPF_6 additive: [KPF_6] = 1.0 mM

Titrant: [TBAX] = 50 mM, X^- = Cl^-, Br^-, I^-

Direct Studies:

Host: [**1·ChB^{PFP}**] = 1.0 mM

Titrant: [KX] = 25 mM, X^- = Cl^-, Br^-, I^-, PF_6^-

Titrations in 98:2 CD_3CN/D_2O (v/v)

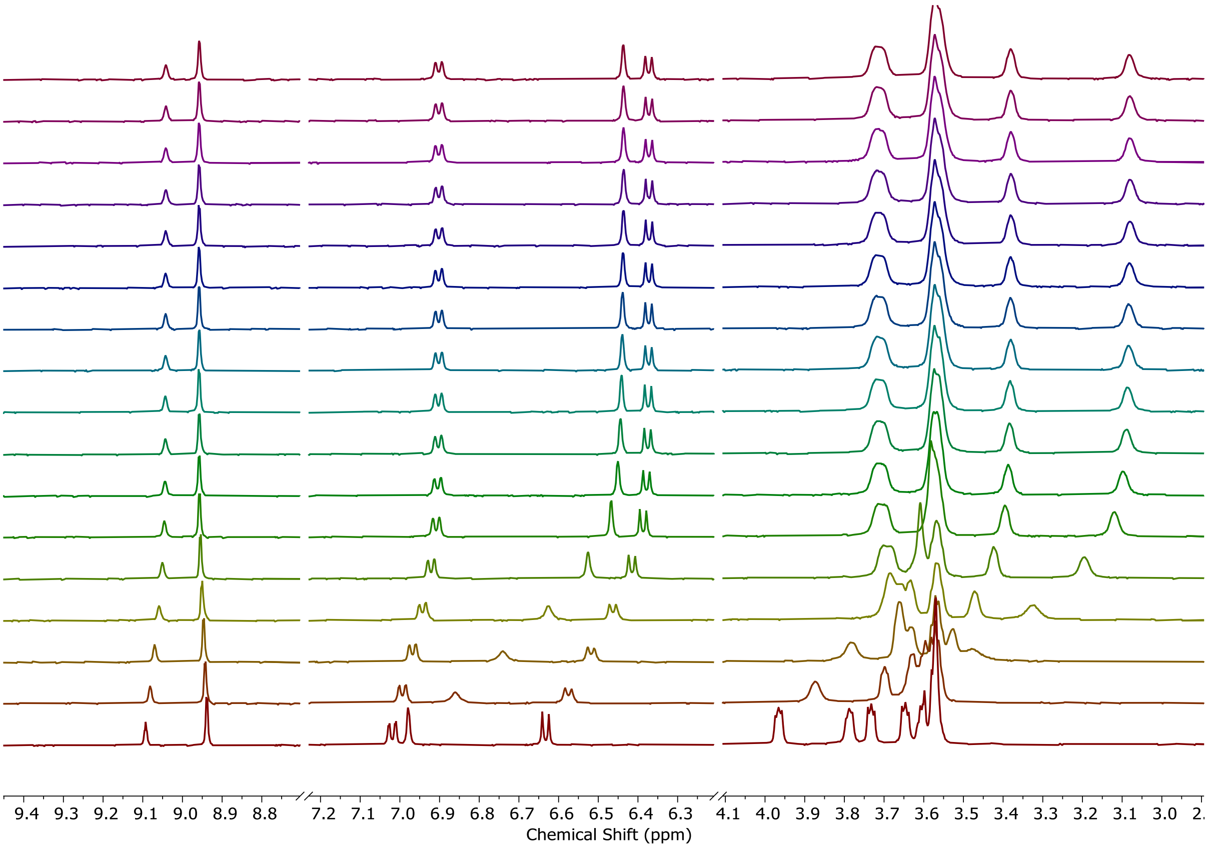

Figure S1. Stacked ^{1}H NMR titration spectra of **$1{\cdot}ChB^{PFP}$** (1 mM) with KPF_6 (25 mM) (98:2 CD_3CN/D_2O (v/v), 500 MHz, 298K).

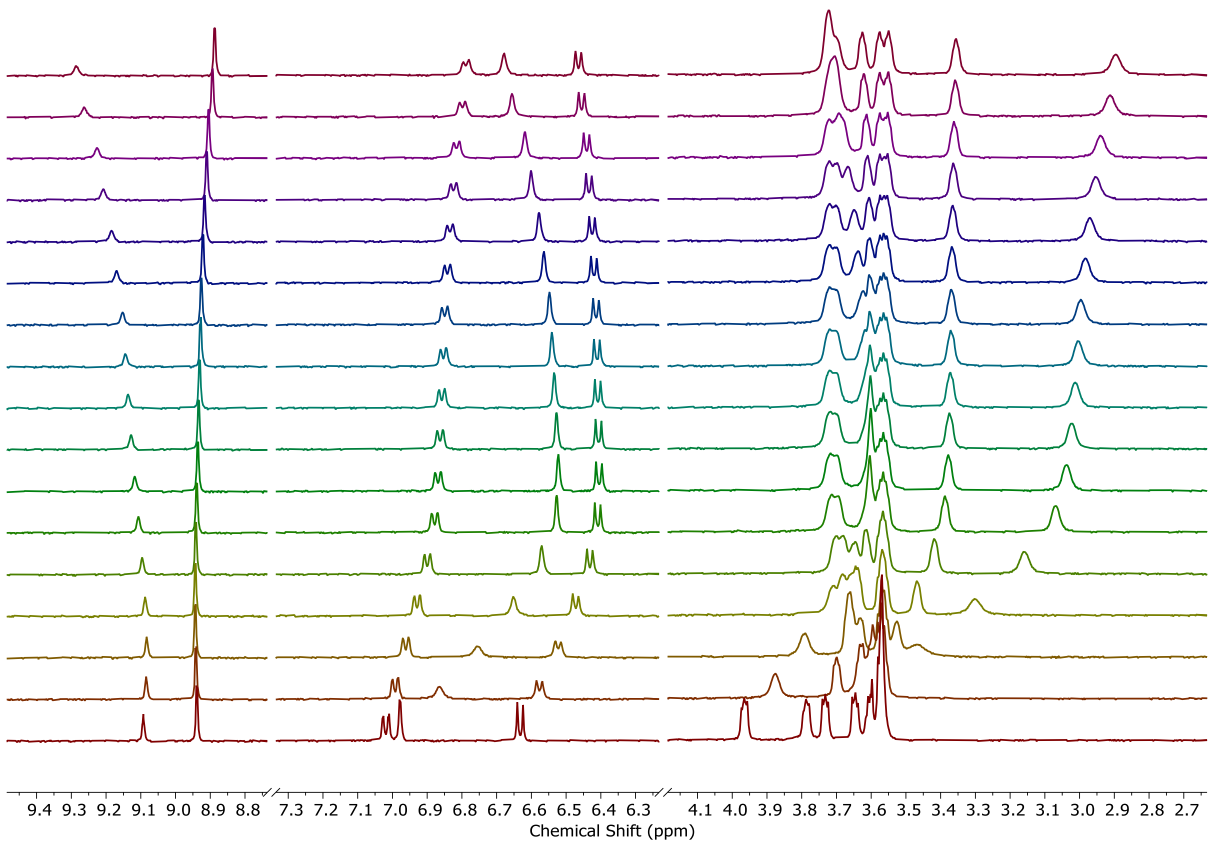

Figure S2. Stacked ^{1}H NMR titration spectra of **1·ChB^{PFP}** (1 mM) with KI (25 mM) (98:2 CD_3CN/D_2O (v/v), 500 MHz, 298K).

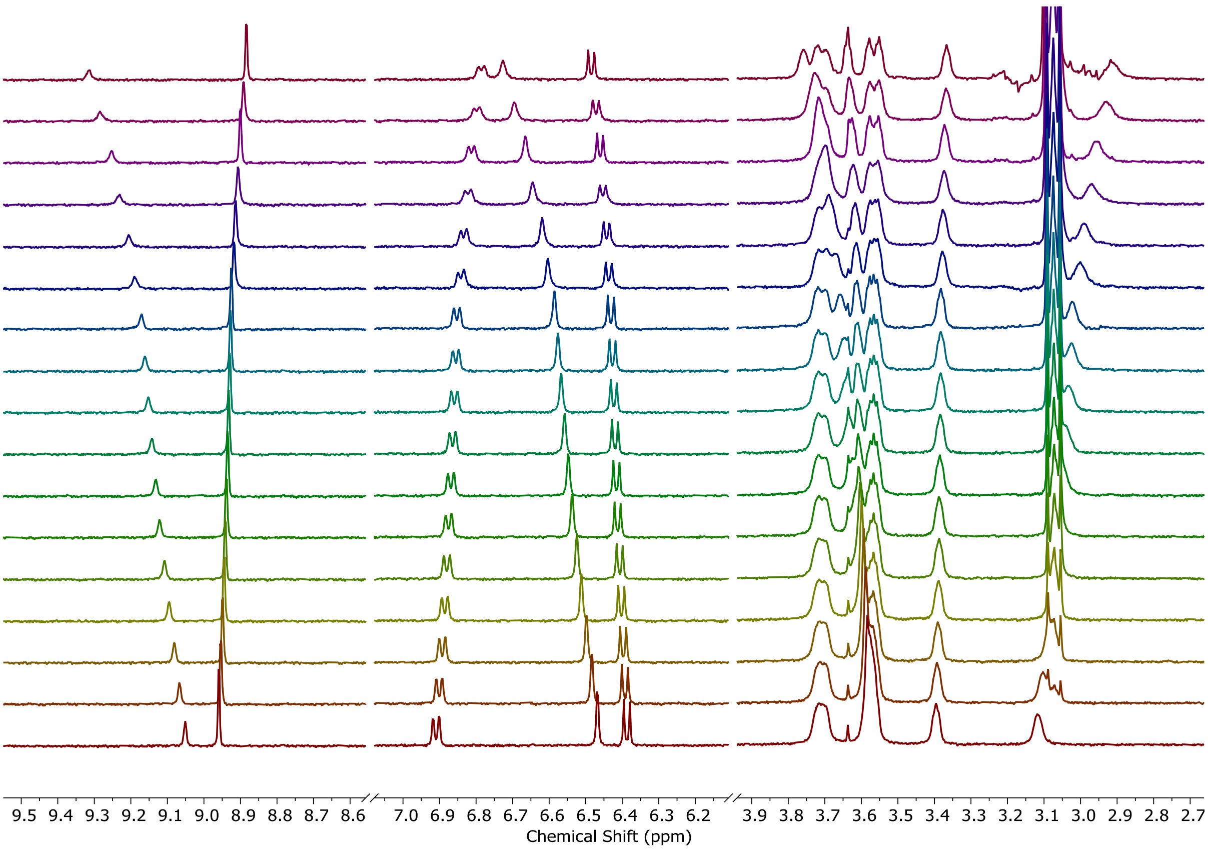

Figure S3. Stacked ^{1}H NMR titration spectra of **1·ChB^{PFP}** (1 mM) with TBAI (50 mM) in the presence of 1 equivalent of KPF_6 (98:2 CD_3CN/D_2O (v/v), 500 MHz, 298K).

Titrations in 90:10 CD_3CN/D_2O (v/v)

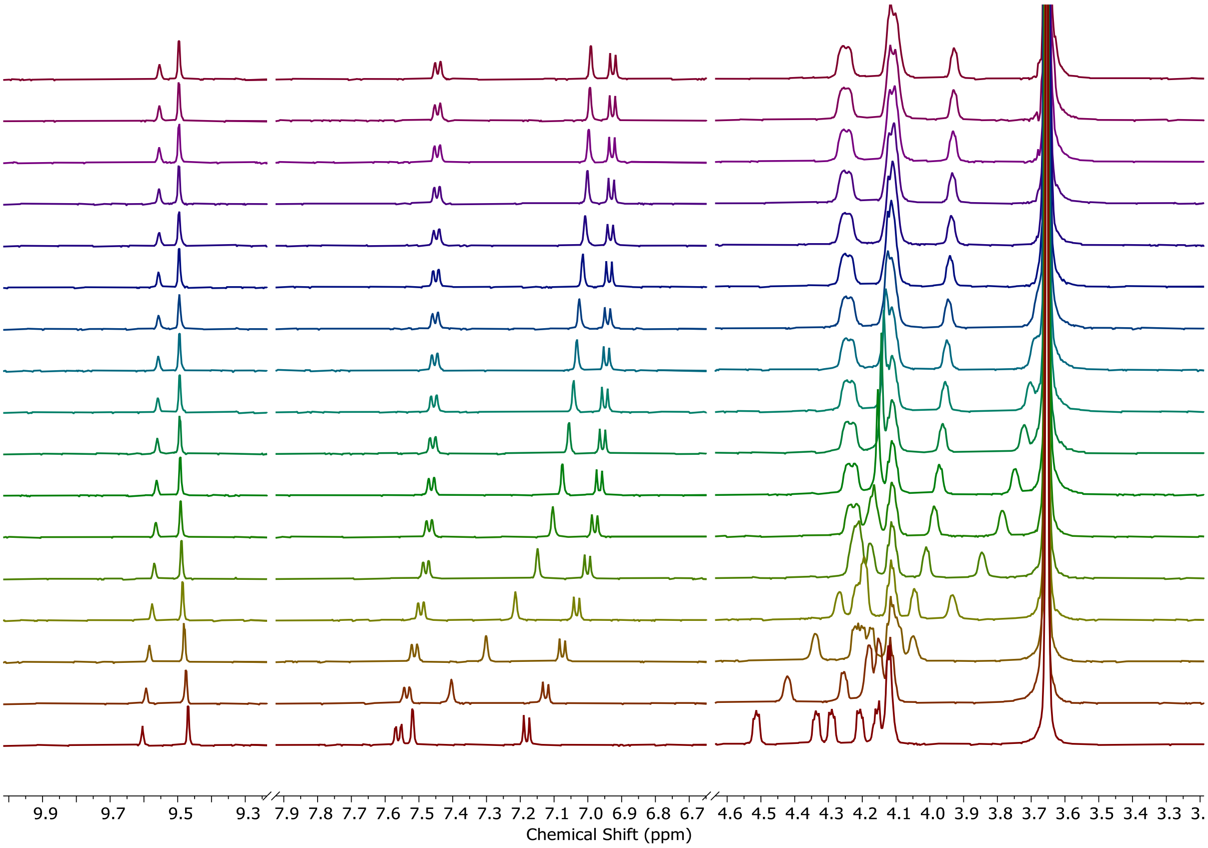

Figure S4. Stacked ^{1}H NMR titration spectra of **1·ChB^{PFP}** (1 mM) with KPF_6 (25 mM) (90:10 CD_3CN/D_2O (v/v), 500 MHz, 298K).

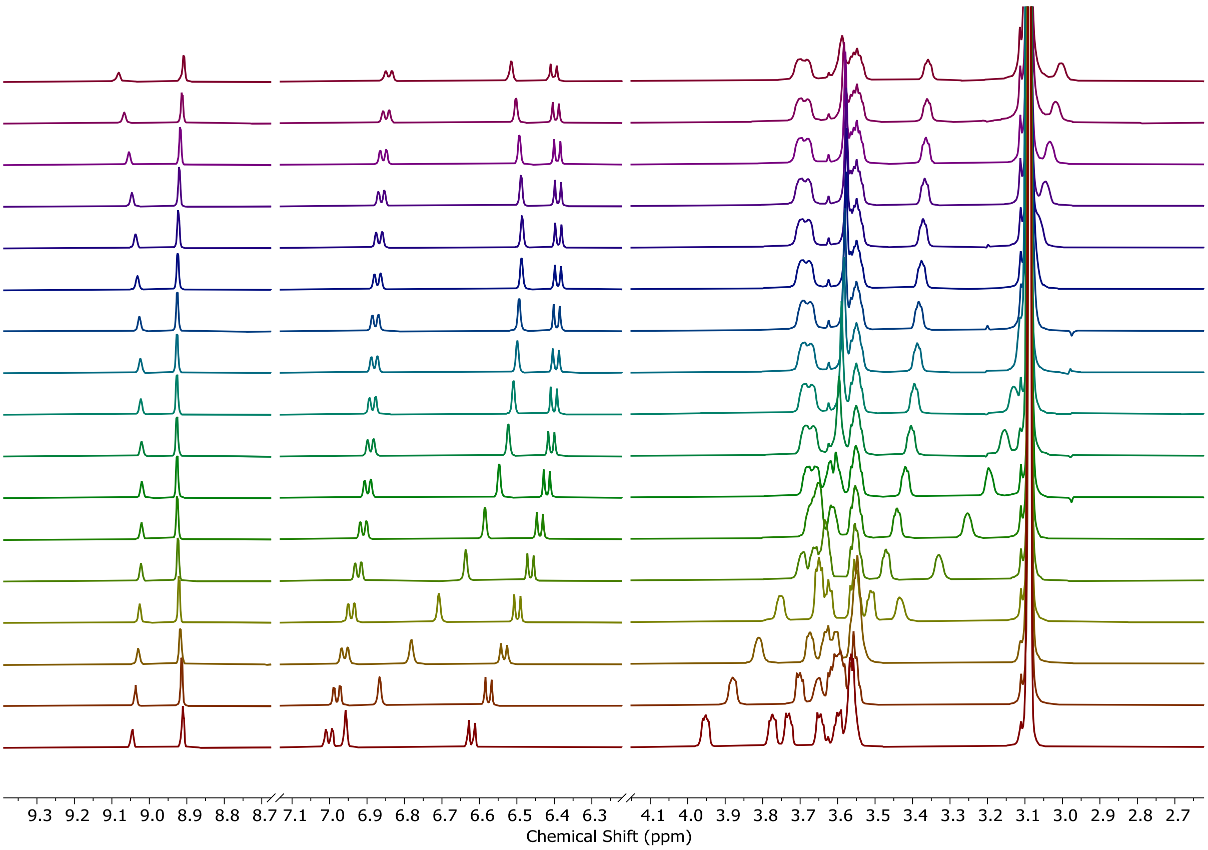

Figure S5. Stacked ^{1}H NMR titration spectra of **1·ChB^{PFP}** (1 mM) with KI (25 mM) (90:10 CD_3CN/D_2O (v/v), 500 MHz, 298K).

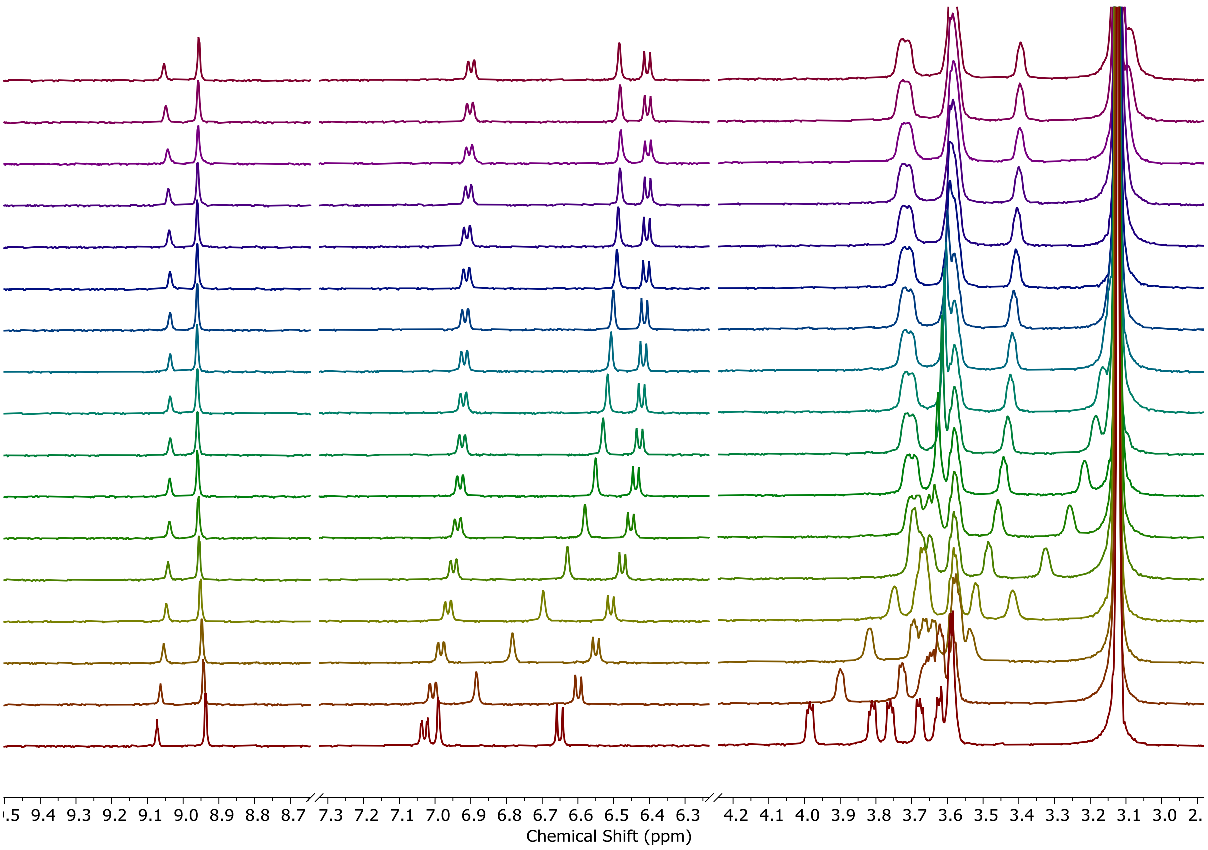

Figure S6. Stacked ^{1}H NMR titration spectra of **1·ChB**$^{\mathbf{PFP}}$ (1 mM) with KBr (25 mM) (90:10 CD_3CN/D_2O (v/v), 500 MHz, 298K).

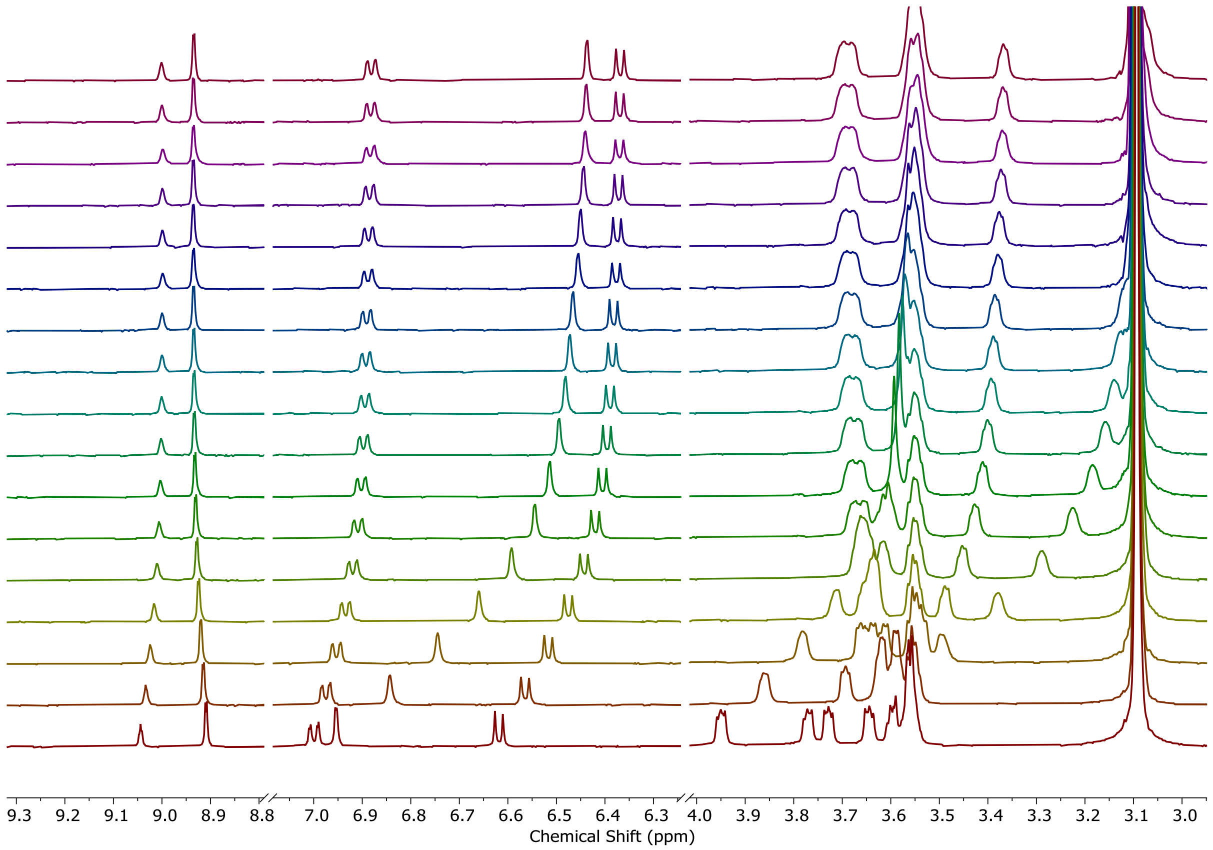

Figure S7. Stacked ^{1}H NMR titration spectra of **1·ChB^{PFP}** (1 mM) with KCl (25 mM) (90:10 CD_3CN/D_2O (v/v), 500 MHz, 298K).

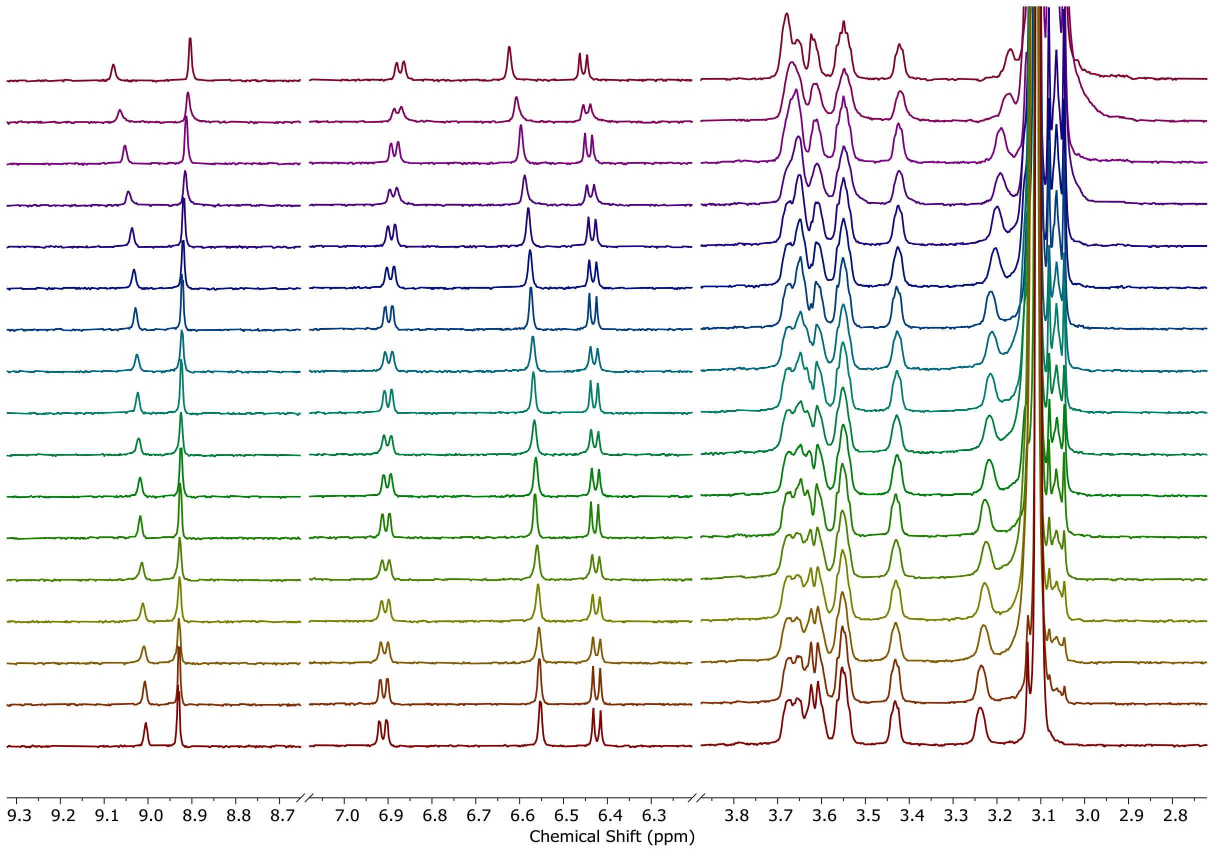

Figure S8. Stacked ^{1}H NMR titration spectra of **1·ChB^{PFP}** (1 mM) with TBAI (50 mM) in the presence of 1 equivalent of KPF_6 (90:10 CD_3CN/D_2O (v/v), 500 MHz, 298K).

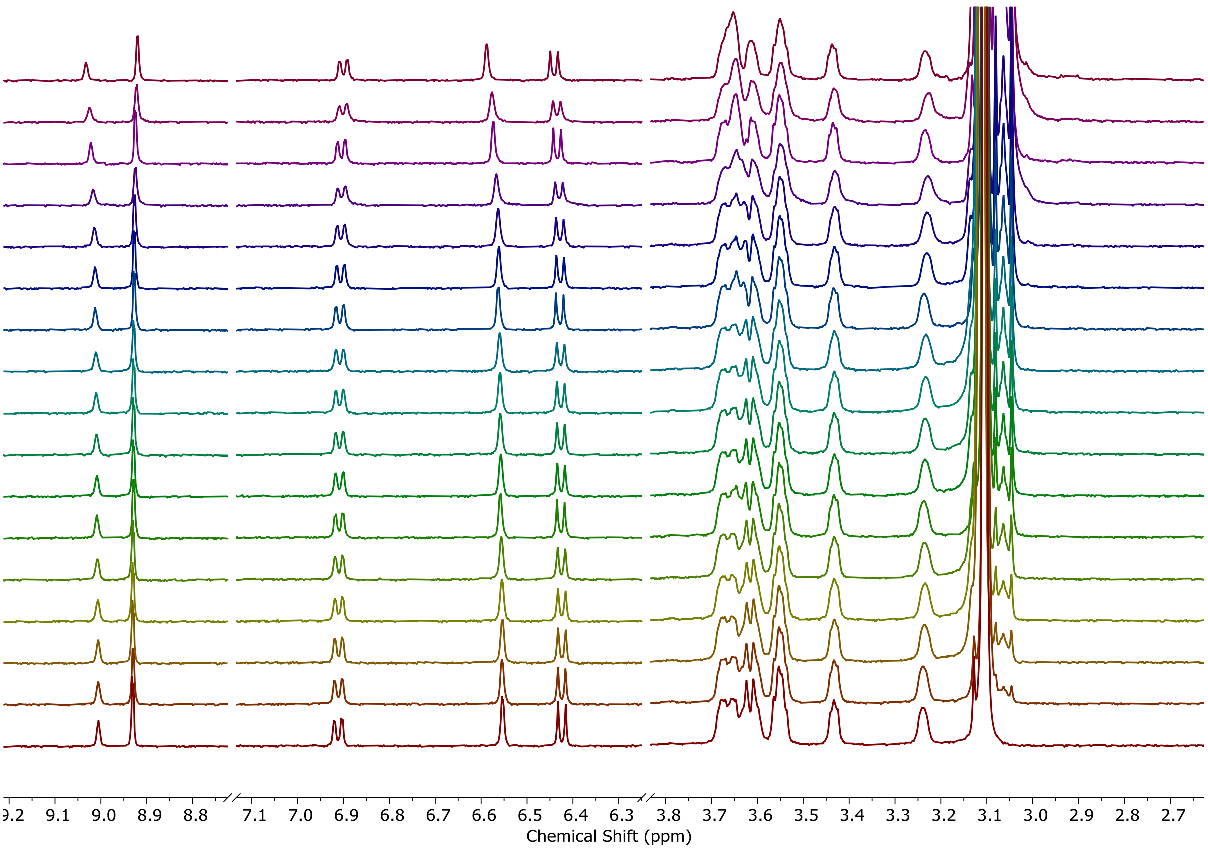

Figure S9. Stacked ^{1}H NMR titration spectra of **1·ChB^{PFP}** (1 mM) with TBABr (50 mM) in the presence of 1 equivalent of KPF_6 (90:10 CD_3CN/D_2O (v/v), 500 MHz, 298K).

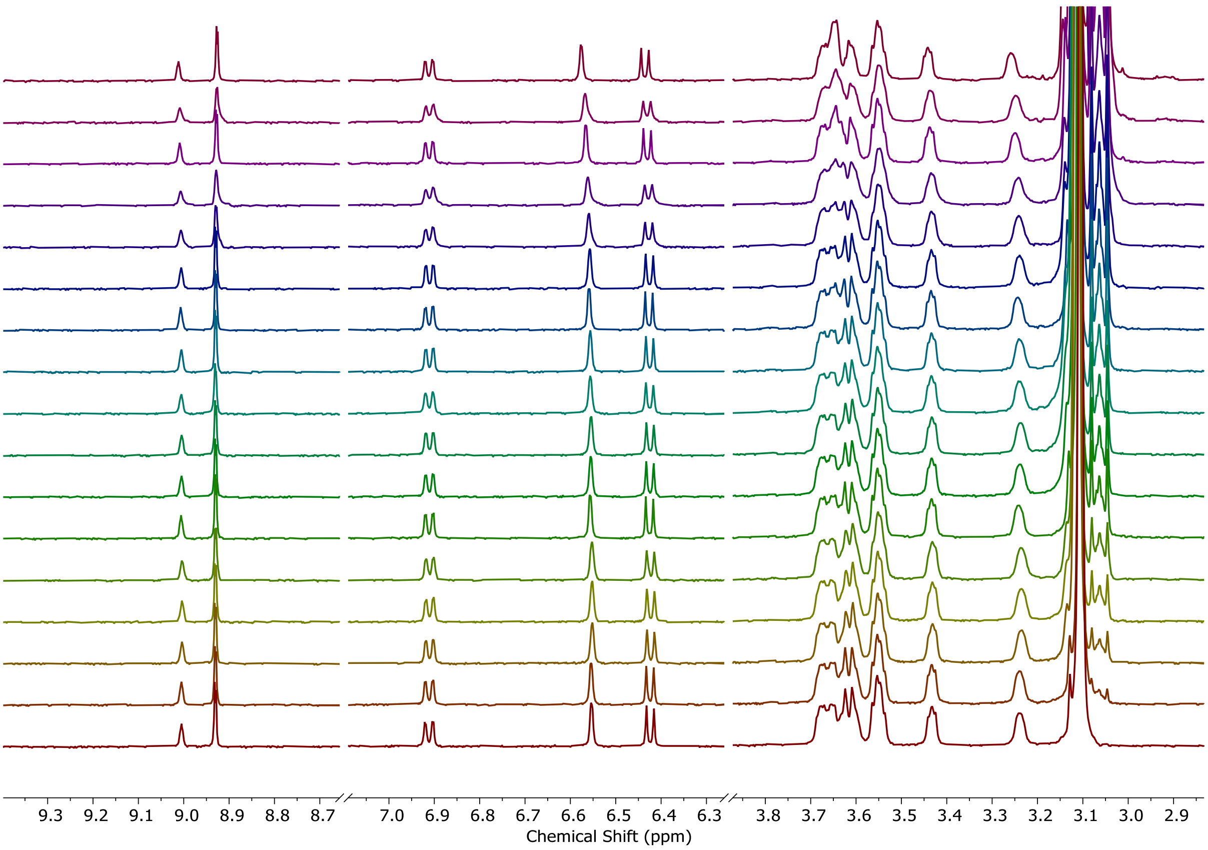

Figure S10. Stacked ^{1}H NMR titration spectra of **1·ChB^{PFP}** (1 mM) with TBACl (50 mM) in the presence of 1 equivalent of KPF_6 (90:10 CD_3CN/D_2O (v/v), 500 MHz, 298K).

3. References

[1] A. Docker, I. Marques, H. Kuhn, Z. Zhang, V. Félix, P. D. Beer, *J. Am. Chem. Soc.* **2022**, *144*, 14778–14789.
